# Experimental Susceptibility of Gilthead Sea Bream, *Sparus aurata,* via Challenge with *Anisakis pegreffii* Larvae

**DOI:** 10.1155/2013/701828

**Published:** 2013-07-11

**Authors:** Fabio Marino, Giovanni Lanteri, Annamaria Passantino, Carmelo De Stefano, Antonella Costa, Gabriella Gaglio, Francesco Macrì

**Affiliations:** ^1^Department of Veterinary Sciences, Centro di Ittiopatologia Sperimentale della Sicilia, University of Messina, Polo Universitario dell'Annunziata, 98168 Messina, Italy; ^2^Istituto Zooprofilattico Sperimentale della Sicilia, Via G. Marinuzzi 3, 90129 Palermo, Italy

## Abstract

The endoscopic and histopathological findings detected in *Sparus aurata* experimentally infected with third-stage *Anisakis* larvae without intermediate host are evaluated and discussed. In six fish, live nematode larvae were introduced by gastroscopy into the stomach. The first observation by endoscope, 15 days after challenge, showed the presence of some larvae at the level of gastric mucosa. An explorative celioscopy, performed 60 days after challenge, showed haemorrhages and/or nodules on the gut of two fishes. Necropsy and histology demonstrated parasites in the context of the tissue changes. The finding of live nematode larvae as well as the evidence of tissue change confirm the experimental susceptibility of gilthead sea bream towards *Anisakis*.

## 1. Introduction

Anisakiasis is a worldwide diffuse fish-borne zoonosis due to ingestion of raw, undercooked, or improperly processed food parasitized by the nematode larval stages of the genus *Anisakis*. In the Mediterranean area, this disease can be considered the most important hazard for human consumers due to ingestion of raw marine fish and cephalopods. The symptoms in human may include diarrhoea, vomit, and abdominal pain, and the main change evoked is an eosinophilic granuloma. Even when thoroughly cooked, *Anisakis* pose a health risk to humans because it releases a number of antigens into the surrounding tissues which can create serious allergic reactions [1].

The etiology of such parasitic disease is linked to nematodes belonging to different genera of the family Anisakidae. Marine mammals (cetaceans, pinnipeds) and fish-eating birds can be definitive hosts, whereas several marine invertebrates, teleosteans, and cephalopods act as intermediate and/or paratenic hosts. 

Although many evidences clarified the pathogenesis of human disease by eating raw fish, few data demonstrate the possibility of experimental transmission of the nematode from a fish to another using *Anisakis* sp. third-stage larvae (L3) without intermediate host [2–7]. 

Imaging techniques have been recently used in teleost fish [8, 9] to assess reproductive stage, although their application in parasitology is not very common. 

The gilthead sea bream is one among the most common fish in Mediterranean aquaculture in which *Anisakis* spp. have never been reported due to its diet. For this reason, this teleost species was selected for this study because they surely cannot be already naturally infected with nematodes.

The aim of this paper is to demonstrate by challenge the susceptibility of the gilthead sea bream towards the nematode worms *Anisakis *sp. and to evaluate and discuss tissue damage due to such larvae in experimentally infected fish by imaging and histopathological techniques.

## 2. Material and Methods

Six (6) gilthead sea bream (*Sparus aurata*) (Linnaeus, 1758), 40–60 g in weight and 9–12 cm in length, were maintained in an aerated 650 l tank, at the Centro di Ittiopatologia Sperimentale della Sicilia (CISS) of the University of Messina, for 60 days long acclimatization period, temperature 25°C, and salinity 35‰. The challenge was performed by endoscopy, using 10 alive *Anisakis* larvae introduced into the stomach of each fish. Larvae were obtained by the contemporaneous necropsy of three teleost fish belonging to the species *Lepidopus caudatus* (Euphrasen, 1788). The study—conducted in accordance with the Directive 2010/63/EU on the protection of animals used for scientific purposes—was performed with the following equipment: a Veress needle (Karl Storz, Tuttlingen, Germany), diameter 2 mm, length 140 mm, and equipped with on-off luer-lock adaptor connected to a standard aquarium air pump; a rigid forward-oblique telescope (Karl Storz), 30° of 1.9 mm in diameter and 19 cm in length, with incorporated fibre optic light transmission, inserted in a cystoscope sheath 10 French (Fr.) in diameter and with a working length of 14 cm, supplied with a 3 French instrument channel, 2 luer-lock cones used for insufflations and aspirations and a trocar; a camera head with lens focus 38 mm (Vet C-Mount, Pal, Karl Storz) connected to a “TELE PACK” device (Karl Storz) providing light source, “LCD monitor 12” and a PCMCIA card slot for digital image capture and storage; a biopsy and grasping forceps (Karl Storz), double-action jaws, flexible, 3 French, 28 cm in length, to collect the bioptic samples.

The fish were anesthetized in a tank containing tricaine methanesulfonate, (MS-222, Sandoz, Switzerland) 0.2 mg/L, and then placed on a grid out of the water and covered with wet gauzes in order to avoid skin lesions. Anaesthesia was maintained conveying water containing MS-222 0.3 mg/L through a small aspiration pump in the oral cavity of the fish, using a flow regulator; water was never recycled. 

Gastroscopy was carried out placing fish on their lateral sides. The endoscope was introduced into the mouth of the fish, driving the extremity towards the oesophagus, until it reached the stomach lumen. Then, *Anisakis* larvae were introduced, using a grasping forceps, through the accessory channel of the instrument and released within the stomach. After verifying vitality of nematodes, the endoscope was removed, and fish were recovered. Another challenge with the same protocol was performed 15 days later. 

After 60 days period, in which fish were daily monitored, an explorative celioscopy, using a rigid cystoscope, was carried out. Celioscopy was carried out placing fish on lateral recumbence; the abdominal region was surgically disinfected with iodopovidone solution. First step was the establishment of pneumocoelom to separate the abdominal wall from the underlying viscera, obtained through a Veress needle, connected to a standard aquarium air pump, inserted into the coelom 1 cm cranially to the anal pore. Then, 8 mm incision was effected with a scalpel 1 cm cranially to the needle, to insert the cystoscope sheath/trocar assembly into the insufflate coelom, by a single firm thrust-and-twist movement. After the entry into the coelom, the trocar was removed and replaced with the telescope to visualize the abdominal cavity. Fish were carefully examined for *Anisakis* larvae and if necessary, bioptic samples were collected.

After the exam, the coelom was air emptied and treated with Enrofloxacin (Baytril, Bayer Animal Health, Leverkusen, Germany) 14 mg/kg; the laparoscopic entry sites were sutured with 5–0 vicryl (Ethicon, Piscataway, New Jersey, USA). The fish were then placed again in a tank with clean water since complete recovery. 

The biopsy samples were fixed in buffered formalin (10%) and paraffin-embedded. 3-4 *μ*m slices were stained with hematoxylin-eosin for routines histology. 

Necropsy was performed 1 week later, and all organs and tissues were sampled for histopathology. On the studied fish, a parasitological exam was performed examining the coelomic cavity for nematode larvae, by visual and stereoscopic evaluation. Parasites sampled from *L. caudatus* and *S. aurata* were isolated, fixed in 70% ethanol, morphologically studied by light microscope, after clarification with lactophenol. 

Tissue samples obtained at necropsy from all organs and tissues of the six teleosts were fixed in 10% buffered formalin solution, rinsed in tap water, rewashed in graded alcoholic solutions, clarified with xylene, and routinely paraffin embedded. 5 *μ*m thick sections were obtained at the microtome and stained with haematoxylin-eosin. 

Four larvae (two from naturally infected *L. caudatus* and two from experimentally infected *S. aurata*), fixed in 70% ethanol and cleared in glycerol, were morphologically identified at genus level by light microscopy and at species level by PCR-RFLP on the basis of the diagnostic restriction banding patterns. In the biomolecular analysis, the larvae were subjected at the DNA extraction and following PCR, by using two primers to amplify the genomic region of the ribosomal DNA, containing ITS1, 5.8S gene, and ITS2 [10]. Amplicons were subjected to restriction analysis with the restriction enzymes *Hha*I, *Hinf*I, and* Taq*I used in RFLP analysis of rDNA, for the identification of the *Anisakis *species. RFLP data have been analysed by electrophoresis on gel revealing restriction models typical for the species, following the keys indicated in literature [11].

## 3. Results

**Figure 1:**
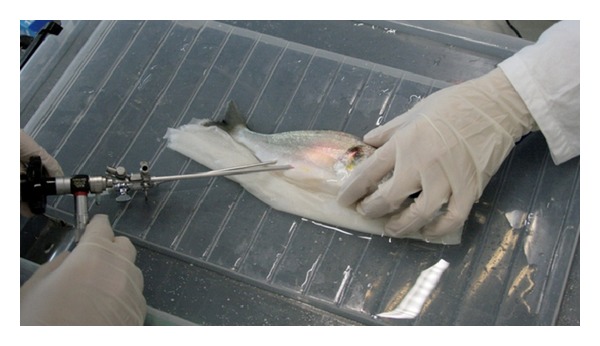
Image of the challenge, celioscopy.

**Figure 2:**
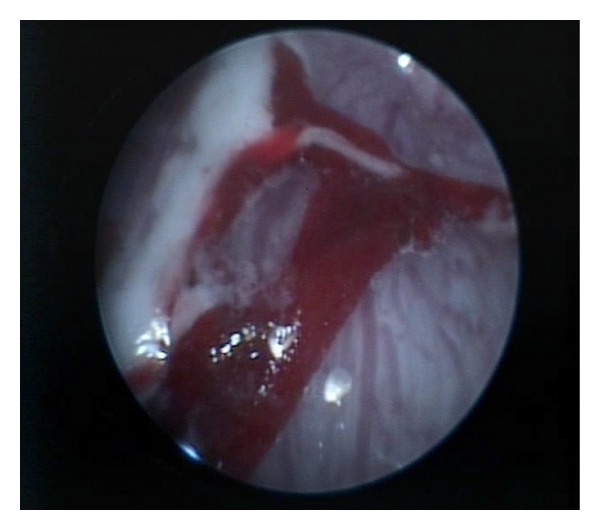
Celioscopy showed a serosal nodular haemorrhagic change from which a single worm arises.

At the time of the second gastroscopic exam (explorative investigation performed 15 days after the challenge) in three fish, some live nematode larvae were found within the lumen of the stomach. At celioscopy (Figure 1), two gilthead seabream (2/6) (33.3%) showed hemorrhages and/or irregular neoformations within the coelomic cavity: one fish had a nodular change with haemorrhage on the serosal surface of the stomach, as well as on the visceral surface of the liver; from the nodule a single nematode larva arised (Figure 2); the second fish had a sharp involvement of the intestinal serosa with different haemorrhages.

At necropsy, the entire coelomic cavity was carefully investigated in all fish. Only two diseased fish were confirmed to have signs of the parasites, as already showed by laparoscopy. Within or close to each tissue change a single nematode larva was detected. 

Histopathology clarified the nature of the tissue changes due to the nematode larvae. These last were identified as hemorrhagic lesions with a cystic aspect. The wall of the cysts were characterized by a mild granulomatous reaction with sheets of macrophages and fibroblasts.

Parasites were morphologically identified by light microscope, as larval forms of the genus *Anisakis* in both *L. caudatus* and *S. aurata*. The larvae were identified as L3 stages, belonging to the morphotype named* Anisakis *Type I (*sensu* Berland 1961). *Anisakis* larvae isolated were identified at species level using the PCR-RFLP techniques: on the basis of the diagnostic restriction banding patterns, PCR-RFLP analysis has allowed to identify larvae isolated from both *L. caudatus* and *S. aurata* as *A. pegreffii* [12].

## 4. Discussion

The present data demonstrate for the first time the susceptibility of the gilthead sea bream towards *Anisakis *spp. and a further report of an experimental infection monitored by endoscopy.

Gastroscopy permits us to introduce the live larvae during the challenge avoiding trauma from mastication, whereas the continuous monitoring of the larval migration from the gastric lumen, throughout the walls of the alimentary tract and the visceral cavity is made by celioscopy. 

The evidence of tissue change is an unusual finding in *Anisakis *infected teleost fish. The finding of haemorrhages could be related to the acute stage of the infection and is in agreement with findings described by Beck et al. [12] and those demonstrated by Macrì et al. [7] in an experimental study on Anisakiasis in the sea bass. The change here described could be explained with the evident susceptibility of the gilthead sea bream towards this parasite. 

*Anisakis* spp. have never been reported to date in wild gilthead sea bream from Mediterranean Sea. Thus, the susceptibility of the gilthead sea bream is here demonstrated for the first time, at least by the experimental point of view. 

Anisakiasis have never been reported in farmed fish, and modern husbandry methods provide warranty of no risk for public health actually derivable from consumption of farmed gilthead sea bream. According to the UE regulations and considering the results here obtained, the use of fresh food in aquaculture must be maintained absolutely forbidden.

Considering that point D. 1 of Chapter III of Section VIII of Annex III to Regulation (EC) no 853/2004 lists the fishery products that must be frozen to a temperature of not more than −20°C in all parts of the product for not less than 24 hours; it is interesting that the legislator inserts this teleost species.

It is also relevant to carry out further studies regarding farmed *Sparus aurata* in order to determine whether it is feasible to recognize the fishery products as being free or not of parasites that might result in a risk to health of the consumers, although no evidences confirming this risk are available in the literature.

In fact the success of control and legislation measures against any parasitic disease is dependent on knowledge of its aetiology and natural history (epidemiology). Whatever the method of control employed, the use of accurate diagnostic tools and studies on the population ecology of parasites can help when elaborating methods of control for fish nematodes.

This study let us to repeat the challenge already performed on Mediterranean sea bass [7], improving the technique regarding times and quality of the surgical approach, and also carefully considering animal welfare. Moreover, the paper can be considered an example of the application of innovative imaging diagnostic tools in fish, which represents an important goal for researchers and veterinarians involved in the field, providing a model to study parasitic infection in fish.
